# Molecular characterization, expression pattern and immunologic function of *CD82a* in large yellow croaker (*Larimichthys crocea*)

**DOI:** 10.3389/fimmu.2024.1301877

**Published:** 2024-02-02

**Authors:** Yue Liu, Yulin Bai, Sijing Chen, Fei Pu, Yaxian Li, Hongshu Chi, Zaiyu Zheng, Peng Xu, Tao Zhou

**Affiliations:** ^1^ State Key Laboratory of Mariculture Breeding, College of Ocean and Earth Sciences, Xiamen University, Xiamen, China; ^2^ Biotechnology Institute, Fujian Academy of Agricultural Sciences, Fuzhou, Fujian, China; ^3^ Fujian Key Laboratory of Genetics and Breeding of Marine Organisms, College of Ocean and Earth Sciences, Xiamen University, Xiamen, China

**Keywords:** *Larimichthys crocea*, *Pseudomonas plecoglossicida*, *CD82a*, apoptosis, antigen presentation

## Abstract

Visceral white spot disease (VWND) caused by *Pseudomonas plecoglossicida* poses a major threat to the sustainable development of large yellow croaker (*Larimichthys crocea*) aquaculture. Genome-wide association analysis (GWAS) and RNA-seq research indicated that *LcCD82a* play an important role in resistance to visceral white spot disease in *L. crocea*, but the molecular mechanism of *LcCD82a* response to *P. plecoglossicida* infection is still unclear. In this study, we cloned and validated the Open Reading Frame (ORF) sequence of *LcCD82a* and explored the expression profile of *LcCD82a* in various tissues of *L.crocea*. In addition, two different transcript variants (*LcCD82a-L* and *LcCD82a-S*) of *LcCD82a* were identified that exhibit alternative splicing patterns after *P. plecoglossicida* infection, which may be closely related to the immune regulation during pathogenetic process of VWND. In order to explore the function of *LcCD82a*, we purified the recombinant protein of *LcCD82a-L* and *LcCD82a-S*. The bacterial agglutination and apoptosis function analysis showed that *LcCD82a* may involve in extracellular bacterial recognition, agglutination, and at the same time participate in the process of antigen presentation and induction of cell apoptosis. Collectively, our studies demonstrate that *LcCD82a* plays a crucial role in regulating apoptosis and antimicrobial immunity.

## 1 Introduction

Large yellow croaker (*Larimichthys crocea*) is an important economic fish in China, and the huge market promotes the rapid development of the large yellow croaker farming industry (1). However, the highly intensive farming model has resulted in frequent occurrence of diseases including VWND caused by *Pseudomonas plecoglossicida* (2). *Pseudomonas* are a group of conditional pathogens, and the health status of the host and the surrounding environmental conditions (such as temperature) are the main factors affecting the pathogenicity of *Pseudomonas* (3). *Pseudomonas plecoglossicida* is a common pathogen in aquaculture, and can infect hosts such as large yellow croaker (*Larimichthys crocea*) (4), *Plecoglossus altivelis* (5), and *Epinephelus coioides* (6), causing visceral white-nodules disease (VWND). VWND caused by *P. plecoglossicida* has become a limiting factor for the healthy and sustainable development of aquaculture. Most studies on *P. plecoglossicida* have focused on the pathogenic mechanism and key virulence genes. Combination of transcriptomic and proteomic analysis indicated that the genes (such as *PVDS2* and *ICMF*) involved in PVD synthesis and T6SS are involved in virulence of *P. plecoglossicida* (7). Dual RNA-seq in *E. coioides* revealed that *secY* is the key virulence gene for the pathogenicity of *P. plecoglossicida*, deepening the understanding of the pathogenic mechanism of *P. plecoglossicida* (8). Several pathological features for VWND can be observed, including white nodules in internal organs such as the spleen, liver and kidneys, as well as ulcers on the skin surface of infected *L. crocea* (9). Outbreaks of visceral white spot disease in cultured *L. crocea* are usually temperature-dependent (15 - 20°C is the optimal temperature), and the mortality rate of infection is extremely high (70 - 80%), which poses a major threat to the sustainability of the *L. crocea* aquaculture industry (3, 10). Studies have been conducted to investigate genes associated with *P. plecoglossicida* infection (11, 12), however the detailed pathogenesis of VWND and host resistance genes are unknown.

We have elucidated the genetic loci and the immune mechanisms of resistance to *P. plecoglossicida* in *L. crocea* by combined Genome-wide association analysis (GWAS) and RNA-seq analyses, and found that the *CD82a* gene and the p53 pathway play critical roles in VWDN resistance (9). *CD82*, also known as *KAI1*, belongs to the tetraspanin superfamily. Tetraspanins are cell-surface glycoproteins that are widely expressed in eukaryotic cell membranes and structurally comprise four hydrophobic membrane-spanning domains, which divide the protein molecule into a small extracellular loop (SEL), a large extracellular loop (LEL) with a CCG motif and two short intracellular termini (13, 14). Each member of tetraspanins assembles into multimeric complexes at the plasma membrane between or with other protein molecules such as integrins, adhesion molecules, and immunoglobulin superfamilies, thereby regulating various physiological processes such as cell differentiation, cell adhesion, apoptosis, viral infection, and immune responses (15–18).

*CD82* has been demonstrated to have important roles in the immune system, including antigen (Ag) recognition and presentation, cell proliferation and apoptosis (19). Apoptosis is an essential component of normal physiological activity and is essential for the regulation of the cell cycle as well as for the growth, development and maintenance of homeostasis in the individual (20). In particular, apoptosis maintains tissue homeostasis and is therefore also involved in the development of some diseases and immune responses (21, 22). The widely reported role of *CD82* is to inhibit the migration of epithelial cells, endothelial cells and the spread of cancer cells (23). Recently, it has been shown that *CD82* also inhibits the migration of phagocytes, including neutrophils and dendritic cells, and promotes antigen presentation (24, 25). Antigen processing and presentation are complex processes that generate phagosomes, and previous studies have shown that *CD82* is recruited to phagosomes during endocytosis of Gram-negative and Gram-positive bacteria (26). This indicates that *CD82* is closely related to and plays an essential role in anti-bacterial infections.

Tetraspanin proteins have also been identified and characterized in fish and other lower vertebrates. A homologue of *CD82*, named *Lja-CD82-like*, exists in the lamprey *Lampetra japonica* and possesses highly conserved four transmembrane structural domains (27). The results showed that *Lja-CD82-like* was significantly expressed in immune tissues and that *Lja-CD82-like* was delivered in the cell membrane and cytoplasm after antigenic stimulation, suggesting that *Lja-CD82-like* may be involved in the immune defense process. Another gene family study in teleost fishes showed that each fish species possesses a different number of tetraspanin genes, and there was an intron gain during the evolution of the *CD82* gene, which has a higher Ka/Ks value and a faster rate of change. Also, this study showed that *CD82* expression level was differentially regulated in zebrafish, medaka, stickleback, and fugu under OP stress. This suggests that tetraspanin genes, including *CD82* may be involved in regulating the immune response process in fish (28). However, the molecular characterization, expression pattern and immunologic function of *CD82a* in large yellow croaker are still unknown.

In this study, we performed cloning, recombinant expression of *LcCD82a*, explored the subcellular localization of *LcCD82a*, investigated the tissue distribution of *LcCD82a* and identified two different transcript variants, and initially characterized the function of *LcCD82a* in apoptosis and *P. plecoglossicida* infection. The results of this study will be helpful for understanding the molecular mechanism of *LcCD82a* in the immunoregulation of VWND in *L. crocea*.

## 2 Materials and methods

### 2.1 Ethics statement

This work was approved by the Animal Care and Use Committee at the College of Ocean and Earth Sciences, Xiamen University. All the methods used in this study were carried out following approved guidelines.

### 2.2 Fish treatment, and sample collection

Healthy large yellow croaker purchased from Fufa Aquatic Breeding Company (Ningde, Fujian) were cultured temporarily in cement ponds (26 ± 0.2°C) for 15 days and then used for artificial infection experiments. During the temporary culture period, the fish were observed daily to ensure that the experimental fish were not infected with pathogens such as *Cryptocaryon irritans* and *P. plecoglossicid*. Feed at the same time every day and stop feeding one week before the start of the experiment. The experimental fish were randomly and equally distributed to two groups with intraperitoneal injection for *P. plecoglossicid* infection. Liver, spleen, gill and head kidney tissues were collected from 6 fish in the control group at 0 h followed by the same tissue samples as above at 72 h post-infection. All fish were anesthetized with tricaine methanesulfonate (MS-222; Sigma, St. Louis, MO, USA) at a concentration of 10 mg/L before sampling, and samples were snap-frozen in liquid nitrogen and stored at −80°C for subsequent RNA extraction and fluorescence quantification.

### 2.3 Gene cloning of *LcCD82a* and transcript variant identification

Total RNA was extracted from the collected tissues with TRIzol™ Reagent (Invitrogen), and cDNA was synthesized by reverse transcription with PrimeScript RT reagent Kit with gDNA Eraser Kit (TaKaRa). The open reading frame (ORF) sequence of *LcCD82a* was obtained from the *L. crocea* transcriptome data (accession number PRJNA836173), and then PCR amplification was performed with primers *LcCD82a-verification-F/R* (**Table 1**). The PCR products were gel-purified and cloned and then sent to Sangon for sequencing to confirm the correct sequence.

**Table 1:** Primers for sequence validation, plasmid construction and qRT-PCR.

Gene Name	Sequence of primers (5’-3’)	Application
*LcCD82a*-*verification-F*	ATGGGGAAAGGCTGCATGAC	Sequence verification
*LcCD82a*-*verification-R*	TTAGTATTTTGGCACTTTGG	
*LcCD82a- RT-F*	TCATGGGCTTCGGACTGTGG	qRT-PCR
*LcCD82a- RT-F*	CCGACGCCGATCAGGATGTA	
*LcCD82a-LEL-F*	CCGGAATTCCAGAAGGACGTGCTAAATGATGAG	Plasmid construction (prokaryotic)
*LcCD82a-LEL-R*	CCGCTCGAGGAGGAGCCAGCTCTCCACGCTGGC	
*LcCD82a-L-F*	CCGGAATTCATGGGGAAAGGCTGCATGA	Plasmid construction (Eukaryotic)
*LcCD82a-L-R*	CCGCTCGAGGTATTTTGGCACTTTGGTGTAAAT	
*LcCD82a-S-F*	CGCGGATCCATGGGCTTCGGACTGTGGCT	Plasmid construction (Eukaryotic)
*LcCD82a-S-R*	CCGGAATTCGTACTTTGGCACTTTGGTGTAAAT	
*β-actin-F*	AAGCCAACAGGGAGAAGATGAC	qRT-PCR
*β-actin-R*	ACGACCAGAGGCATACA	

### 2.4 Bioinformatics analysis of amino acid sequences

Transmembrane structural domains were analyzed using the SOSUI website (https://harrier.nagahama-ibio.ac.jp/sosui/). Alphafold (v. 2.2.2) was used for protein 3D structure prediction. The shear isoforms of *LcCD82a* were re-annotated using GMAP software, and the structures were visualized using R scripts. SnapGene was used for multiple sequence comparison of sequencing data.

### 2.5 Cell culture and plasmids construction

In subsequent experiments (overexpression, flow cytometry), we explored the biological functions of *LcCD82* using LYCK cell line. The LYCK cell line was a generous gift from the Fujian Academy of Agricultural Sciences. LYC cells were cultured in 199 Medium (M199, Gibco) containing 100U/ml penicillin-streptomycin and 10% fetal bovine serum (FBS, Gibco) at 28°C with 5% CO_2_. HEK-293T cells were cultured in DMEM Medium (Gibco) containing 100U/ml penicillin-streptomycin and 10% fetal bovine serum (FBS, Gibco) at 37°C with 5% CO_2_. In order to express *LcCD82a*, the ORF of *LcCD82a* was linked to the overexpression vector pCMV-flag to construct *pCMV-flag-LcCD82a_L/S*, which can express flag-tagged *LcCD82a_L/S* protein. In addition, we also used a vector with enhanced green fluorescent protein (EGFP) linked to *LcCD82a_L/S*, which can simultaneously express green fluorescent protein and *LcCD82a_L/S* protein, for exploring the subcellular localization of *LcCD82a*. To obtain recombinant proteins, *LcCD82a-LEL* was amplified by PCR using primers *LcCD82a-LEL-F/R*, ligated to *pET32a* vectors.

### 2.6 Subcellular localization

Cells were seeded into 96-well glass bottom plate and approximately 60% confluence at 28°C. Then, 100 ng of *pCMV-EGFP-LcCD82a_L/S* or *pCMV-EGFP* empty vector (control) were transfected into HEK-293T cells using Lipo 8000 Transfection Reagent (Beyotime) following the manufacturer’s protocol. After transfection 48 h, cells were washed with PBS, fixed with 4% paraformaldehyde for 15 min, treated with 0.1% ~ 0.2% Triton-X-100 for 5 min at room temperature, and then stained with DiD and Hoechst for 5 min at room temperature. Finally, the cells were washed five times with PBS and then observed under LSM780 (Carl Zeiss). Each experiment has three independent biological replicates and at least three technical replicates. Each group was repeated three times independently.

### 2.7 Overexpression and flow cytometry assay

LYCK cells were seeded into a 6-well plate, and 2.5 µg of *pCMV-myc-LcCD82a* or *pCMV-myc* empty vector (control) were transfected into LYCK cells using Lipo 8000 Transfection Reagent (Beyotime) following the manufacturer’s protocol. The effect of overexpression was confirmed by qPT-PCR detection of mRNA expression level. Inactivated *P. plecoglossicid* (10^6^ CFU/ml) was added to stimulate the cells after 8 h post-transfection. Cells that were not transfected were also subjected to the same treatment as a control. After 12 hours of treatment, the cells were washed with PBS, and the apoptosis was detected with Annexin V-FITC/PI Apoptosis Detection Kit (Beyotime) in a CytoFLEX (Beckman Coulter Life Science). Each experiment has three independent biological replicates and three technical replicates.

### 2.8 Expression and purification of recombinant protein and bacterial agglutination assay

In order to obtain recombinant *LcCD82a-LEL* protein, use primers F and R to amplify the *LcCD82a-LEL* fragment with XhoI and EcoRI restriction sites, and insert it into *pET-28a* plasmid. Transform *pET-28a-LcCD82a-LEL* into *E. coli*, add IPTG (final concentration 0.5mM) when the transformant grows to OD_600_ = 0.6, and then continue to grow at 28°C for 4 h. Ultrasonic lysis of *E. coli* was performed on ice after replacing the bacterial culture solution with buffer (300 mM NaCl, 50 mM NaH_2_PO_4_, and 10 mM imidazole; pH 8.0). The supernatant was collected by centrifugation and filtered using a 0.22 μm acrodisc syringe filter, then recombinant proteins was eluted using elution buffer (300 mM NaCl, 50 mM NaH_2_PO_4_, and 250 mM imidazole; pH 8.0) after supernatant passing through a Ni-NTA column and dialyzed in PBS at 4°C to eliminate imidazole.

*P. plecoglossicida* and *Staphylococcus aureus* were amplified and cultured at 37°C to the exponential phase (OD_600_ = 0.6), centrifuged at 12000 g for 15 min, and the bacteria were resuspended in PBS to obtain a final bacterial suspension with OD_600_ = 0.2. Add 10 μl of bacterial suspension to 25 μl r*LcCD82a-LEL* dissolved in the same buffer as negative control and incubated at room temperature for 45 min, then observed under confocal microscope and electron microscope. Each experiment has three independent biological replicates and three technical replicates.

### 2.9 Tissue expression profile

RNA was extracted from the collected tissues, and then, according to the protocol of PrimeScript™ RT reagent Kit (Takara, Japan), first-strand cDNA was synthesized using total RNA as a template. Expression patterns and temporal expression changes of *LcCD82a* in response to *P. plecoglossicida* infection were determined by qRT-PCR with primers *LcCD82a-RT-F* and *LcCD82a-RT-R* (**Table 1**). The reference gene is β-actin. The relative expression at each time point was analyzed using the 2^-ΔΔCt^ method. Results were analyzed using one-way ANOVA and plotted using GraphPad Prism software. Each experiment has three independent biological replicates and three technical replicates.

## 3 Result

### 3.1 Sequence and structural characteristics of *LcCD82a*

**Figure 1:**
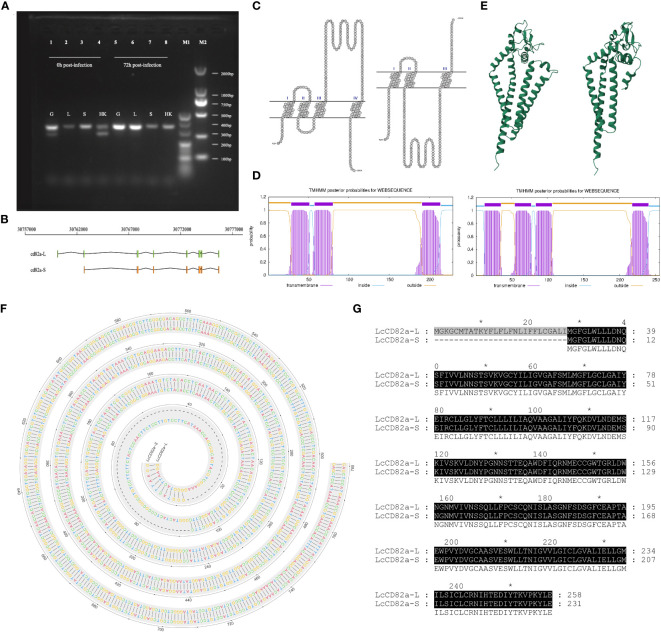
Structural prediction and splicing pattern analysis of transcript variants *LcCD82a-L/S*. **(A)** PCR verification of alternative splicing patterns. **(B)** Linear structures of two transcript variants. **(C)** Visualization of transmembrane structure of transcript variants. **(D)** Three-dimensional structural reconstruction of proteins encoded by transcript variants. **(E)** Prediction of transmembrane regions of proteins encoded by transcript variants. **(F)** DNA sequence alignment of *CD82a-L* and *CD82a-S*. The “-” indicates differences. **(G)** Amino acid sequence alignment of *CD82a-L* and *CD82a-S.* The results of the homology analysis showed 100% sequence homology between *CD82a-L* and *CD82a-S*, except for the first exon region (indicated by black markers in the figure, with darker colors indicating higher homology).

Primers for *LcCD82a* were designed in the conserved regions of the two transcripts, PCR amplification and sequencing were performed using gill, liver, spleen and head kidney tissues sample as a template, and the results showed that transcript variants existed in gills and head kidney (**Figure 1A**), that is, there were two transcripts before infection, and only the transcript with a tetraspanin structure was present after infection. They were named *LcCD82a-L* and *LcCD82a-S* according to their length, the complete transcript length is 334 bp, and the variant is 262 bp. *LcCD82a-S* had a loss of the first exon (**Figure 1B**), and this caused a backward shift of the coding start site (**Figures 1F, G**), thereby changing the post-translational protein structure (**Figures 1C–E**). The visualization of the transmembrane structure, the reconstruction of the three-dimensional structure of the protein, and the prediction of the transmembrane region showed that *LcCD82a-L* has a classic four-transmembrane structure (two extracellular regions, three intracellular regions, and four transmembrane regions). The two extracellular regions were divided into LEL and SEL, but the intracellular segment and SEL structure of the N-terminal of *LcCD82a-S* protein were destroyed.

### 3.2 Expression and purification of recombinant proteins

The bacteria in the fermentation broth were sonicated, the target protein was expressed in its soluble form, and the eluted sample was purer and at a higher concentration when the concentration of imidazole was 250 mM (**Figure S1**). The samples with high purity were mixed to obtain about 5 ml of high-purity *LcCD82a-LEL* protein, and the protein concentration was about 0.33 mg/ml.

### 3.3 Whole tissue and immunological tissue expression profiles of *LcCD82a*

**Figure 2:**
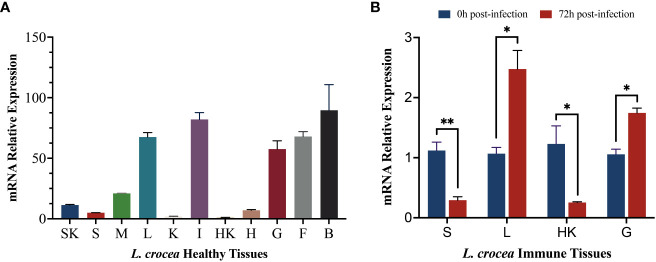
mRNA expression profiles of *LcCD82a* in healthy tissues and immune tissues. The data represents the log2 fold change of expression at different time points compared to the control group. **(A)** Relative mRNA expression levels of *LcCD82a* in various tissues. **(B)** The mRNA expression profiles of *LcCD82a* in immunized tissues at different time points after challenge. *p < 0.05, **p<0.01.

To explore the differences in the expression levels of *LcCD82a* among different tissues, we examined it in 11 tissues of healthy *L. crocea*. Furthermore, the *LcCD82a* gene was quantified in immune tissues before and after infection, to explore whether the infection of *P. plecoglossicida* in immune tissues would cause the differential regulation of *LcCD82a*. The results showed that the *LcCD82a* gene was widely expressed in most tissues of *L.crocea* (**Figure 2A**), with the highest expression in brain (B), intestinal tract (I), and liver (L) tissues, kidney (K), head kidney (HK), and spleen (S) had the lowest expression. After infection with *P. plecoglossicida*, the expression of *LcCD82a* was down-regulated in spleen (S) and head kidney (HK) (**Figure 2B**), which were about one-fifth (26% and 20%) of the pre-infection level respectively. The expression levels in livers (L) and gills (G) were up-regulated, which were 2.32 times and 1.65 times that before infection, respectively, and the differences were significant (P < 0.05).

### 3.4 Subcellular localization of different transcript variants

**Figure 3:**
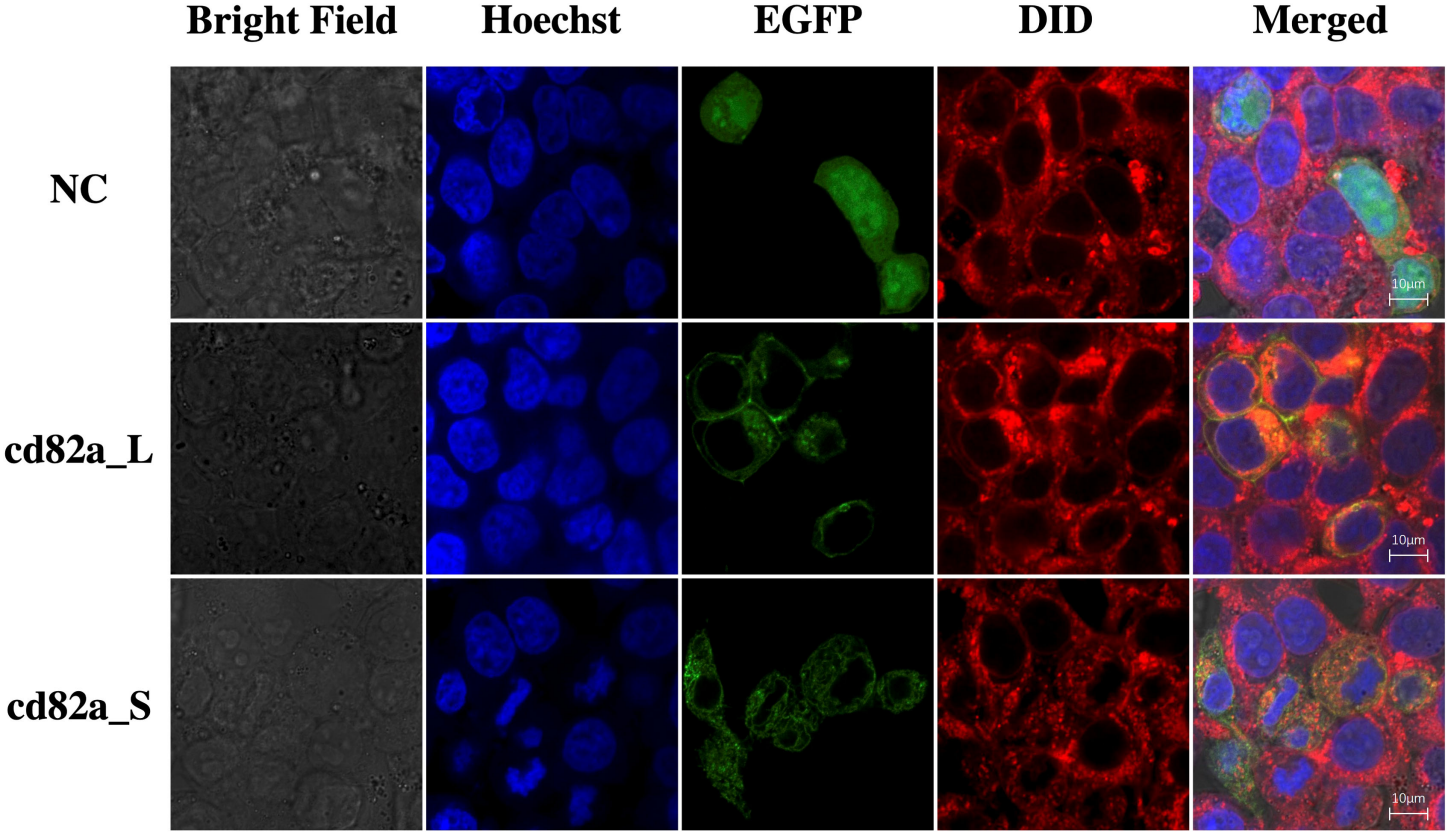
Subcellular localization of transcript variants *LcCD82a-L/S*. The first column is the cell morphology taken in bright field, the second column is the nuclear staining with Hoechst dye, the third column is the fusion protein of the target sequence and EGFP, the fourth column is the cell membrane staining with DID dye, and the last column is the result after merge.

The cell lines were transfected with *pCMV-LcCD82a-S-EGFP* and *pCMV-LcCD82a-S-EGFP* two plasmids with green fluorescent markers for subcellular localization, and the empty plasmid (*pCMV-C-EGFP*) was used as a control group. The fusion proteins of r*LcCD82a-L* and r*LcCD82a-S* share the same distributional location in the cell, being strongly localized to the cell membrane, the nuclear membrane and various organelle membranes (**Figure 3**). This suggests that the structural differences between *LcCD82a-L* and *LcCD82a-S* do not affect the position of distribution in the cell, and that both proteins function on the biomembrane system. The green fluorescent protein of the control group was widely distributed in the nucleus, cytoplasm and cell membrane (**Figure 3**), indicating that the green fluorescent protein and the carrier plasmid did not affect the subcellular localization of the target protein.

### 3.5 Effect of *LcCD82a* on apoptosis

**Figure 4:**
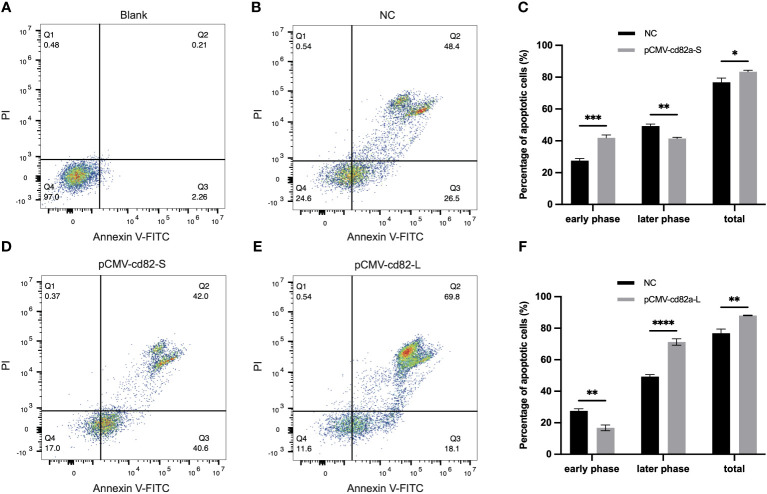
*LcCD82a-L/S* activates apoptosis of LYCK cells stimulated by *P. plecoglossicida.* **(A, B)** Blank control and negative control. **(C, D)** Changes in apoptotic rate of LYCK cells after *LcCD82a-S* overexpression followed by *P. plecoglossicida* challenge. *p < 0.05, **p < 0.01, ***p < 0.001. **(E, F)** Changes in apoptotic rate of LYCK cells after *LcCD82a-L* overexpression followed by *P. plecoglossicida* challenge. **p < 0.01, ***p < 0.001, ****p < 0.0001.

In order to explore the molecular functions of *LcCD82a*, *LcCD82a-L* and *LcCD82a-S* were overexpressed in LYCK cells, which were stimulated with inactivated *P. plecoglossicida*, and then detected by flow cytometry. **Figures 4A, B** show the control groups. The percentage of normal cells in cells transfected with *pCMV-LcCD82a-S* was 17.0% (**Figure 4D**), which was lower than that in the control group (24.6%), and the apoptosis rate was 82.6%, of which 40.6% were early apoptotic cells and 42.0% were late apoptotic cells. The apoptosis rate after transfection of *pCMV-LcCD82a-S* was significantly higher than that of the control group (*P* < 0.05), but the percentage of late apoptotic cells was significantly lower than that of the control group (48.4%), mainly caused by the significantly higher percentage of early apoptotic cells than that of the control group (**Figure 4C**). The percentage of normal cells in cells transfected with *pCMV-LcCD82a-L* was 11.6% (**Figure 4E**), and the apoptosis rate was 87.9%, of which 18.1% were early apoptotic cells and 69.8% were late apoptotic cells. The apoptosis rate after transfection with *pCMV-LcCD82a-L* was higher than that of the control group (74.9%), but the number of early apoptotic cells was reduced, mainly contributed by the number of late apoptotic cells. **Figure 4F** demonstrates that the apoptosis rate of LYCK cells was significantly upregulated after overexpression of *LcCD82a-L* (*P* < 0.01).

### 3.6 Bacterial agglutination activity of *LcCD82a*

**Figure 5:**
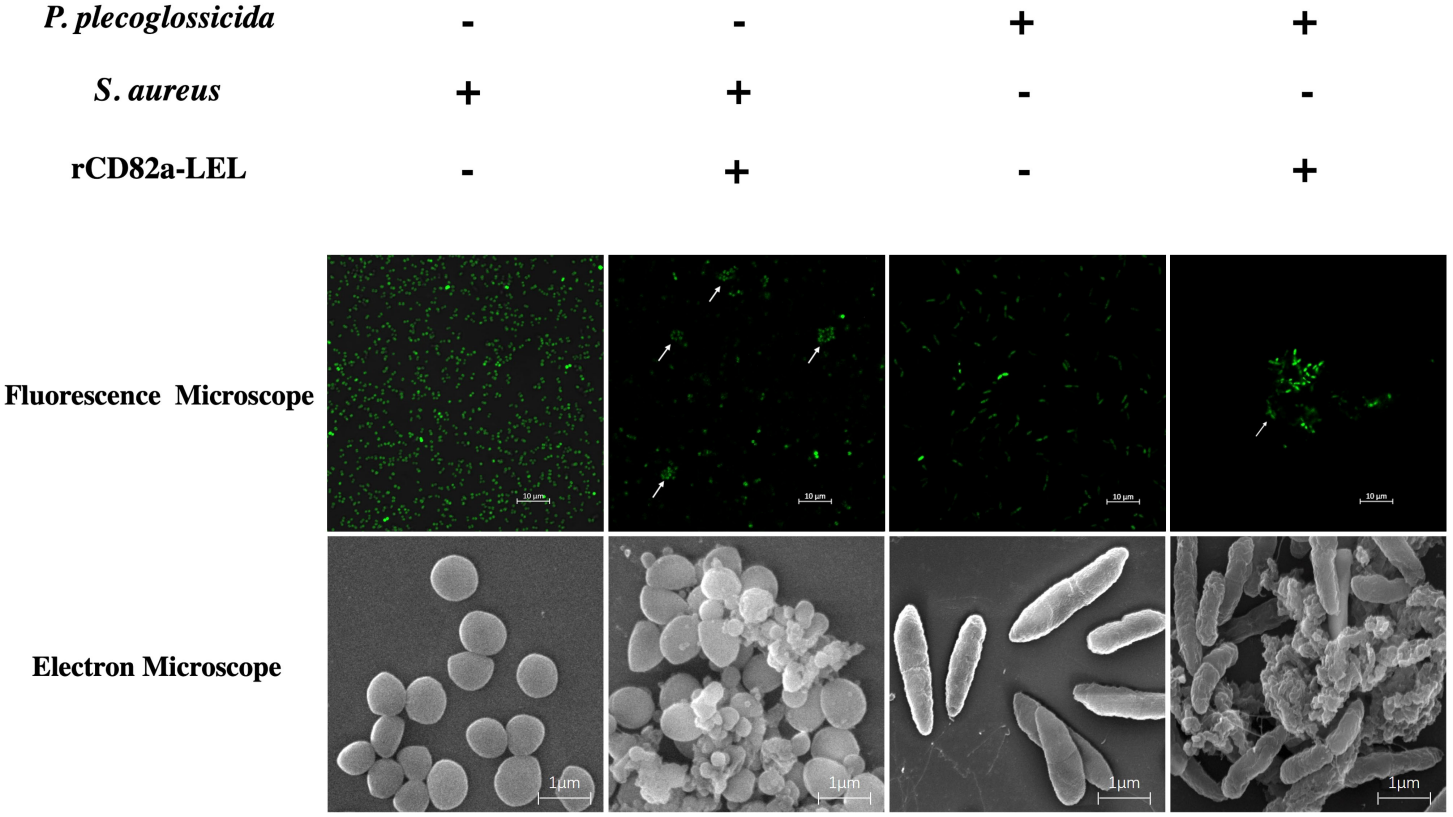
Agglutination activity of r*LcCD82a-LEL* against FITC-labeled Gram-positive bacteria (*S. aureus*) and Gram-negative bacteria (*P. plecoglossicida*). FITC-labeled *S. aureus* and *P. plecoglossicida* were co-incubated with r*LcCD82a-LEL*, and images were taken using an LSM 780 confocal microscope, with arrows showing bacterial agglutination.

In this study, bacterial agglutination assays were performed using r*LcCD82a-LEL* recombinant protein, using *S. aureus* and *P. plecoglossicida* (**Figure S2**) as model pathogens, respectively. Fluorescent labeling was performed on both bacteria, which were photographed using fluorescence confocal microscopy (100×) and electron microscopy (50,000x), respectively. *S. aureus* and *P. plecoglossicida* without recombinant protein added were both evenly distributed in the field of view with regular, intact shapes. After adding r*LcCD82a-LEL*, bacterial agglutination was observed in both *S. aureus* and *P. plecoglossicida* (**Figure 5**). And under the electron microscope, it could be observed that the morphology of the bacteria changed, and some of them ruptured and became wrinkled after death.

## 4 Discussion

As an important factor for maintaining normal physiological activities, *CD82a* is widely distributed in various tissues and is specifically expressed in different physiological and pathological states (29–33). Our results demonstrated that *LcCD82a* was expressed in various tissues of healthy *L. crocea*, with the highest expression level in the brain, intestine, and liver. Previous studies in *Lampetra japonica* have shown similar results, with *CD82* mRNA detected in several tissues and highest in blood cells and liver. It was reported that *CD82* play a significant role in the defense against pathogens such as bacteria (26). In this study, exploration of *LcCD82a* expression patterns in immune tissues during *P. plecoglossicida* infection revealed significant differential expression in all tested immune tissues during the peak infection period (upregulation in liver and gills, downregulation in spleen and head kidney), which was consistent with the pathogen distribution identified using immunohistochemistry (IHC) and confirming the presence of immune cell aggregation in tissues such as liver and gills of *Pseudosciaena crocea* (34, 35).

We characterized two *LcCD82a* gene transcript variants, *LcCD82a-L* and *LcCD82a-S*, with transcript differences primarily located in the first exon region. The shorter transcript variant *LcCD82a-S* lacks a portion of the first exon, resulting in the disruption of the N-terminal cytoplasmic domain and extracellular loop, leaving only the remaining three major transmembrane regions. Moreover, an analysis of differential splicing patterns in gills and head kidney tissues before and after infection indicated selective splicing of *LcCD82a-S* specifically before infection. CD82 has functions such as antigen presentation (19), and the head kidney is an important immune organ that can produce a variety of immune cells, and the gills are rich in blood and leukocyte (36). Therefore, the *LcCD82a-L* may be involved in the immune defense of organisms against bacterial infections, and the coding region of the first exon may be closely related to bacterial infections, antigen recognition and presentation.

Subcellular localization can explore the specific location of a certain protein or expression product distribution in cells, thus providing a research direction for understanding the mechanism of action of genes. We explored the subcellular localization of *LcCD82a* and found that both *LcCD82a-L* and *LcCD82a-S* were localized in the cytoplasm, nuclear membrane, organelle membrane and cell membrane. Previous evidence has shown that *CD82* is a tetraspanin that is ubiquitously present in various biological membranes, such as the inner membrane of exosomes carrying major histocompatibility complex (MHC) class II molecules (37). The subcellular localization of *LcCD82a* provides strong evidence for its function. *LcCD82a* is a member of the tetraspanin family and plays an antigen-presenting function on biological membranes. Its localization in the nuclear membrane and cytoplasm is closely related to apoptosis.

Apoptosis plays an important role in maintaining normal tissue development and homeostasis, and dysregulation of apoptotic signaling can lead to various pathological conditions in organisms. Cell apoptosis is an important pathway limiting pathogen replication and dissemination, and early apoptosis following pathogen infection can contribute to disease control. Therefore, we overexpressed *LcCD82a-L* and *LcCD82a-S* in LYCK cells to investigate the effects of two different transcriptional variants of *LcCD82a* on apoptosis after stimulation by *P. plecoglossicida*. Our results showed that although the two transcript variants affected apoptosis in different ways and degrees, they both promoted apoptosis after pathogen infection in general. Previous research has shown that some viruses have genes that inhibit the apoptosis of infected cells and promote their own survival and proliferation. For example, the proteins encoded by the BHRF-1 gene of Epstein-Barr virus (EBV) (38) and the LMW5-HL gene of African swine fever virus (ASFV) (39) are similar to the BCL-2 protein sequence, so they also play the role of inhibiting apoptosis. In addition, the gene LMP-1 of EBV inhibits p53-triggered apoptosis during the incubation period of infection, creating favorable survival conditions for infected B cells (40). Consequently, *LcCD82a* promoted apoptosis of *P. plecoglossicida*-infected LYCK cells, which could clear the pathogen and inhibit its proliferation to a certain extent, thereby playing a positive role in the process of resisting *P. plecoglossicida* in *L. crocea*. In addition, cells are phagocytized by phagocytic cells after apoptosis, and the cell contents do not flow out without triggering inflammation, which is a relatively mild process (41). *LcCD82a* induces apoptosis in infected cells, which reduces the extent of damage in the diseased area and improves the survival rate of *L. crocea* as compared to inflammation caused by excessive immune response.

Cell apoptosis and antigen presentation processes are interdependent, with apoptotic cells or apoptotic bodies serving as the major source of cross-presentation of antigens (42). Phagocytosis is a primitive defense mechanism, and teleost M1 macrophages are an important component of teleost innate immunity, which can recognize and phagocytose invading pathogens (43–45). In addition, macrophages are also an important bridge connecting the innate immunity and adaptive immunity of teleost. Macrophages present intracellular pathogen-derived antigens to T cells, thereby triggering adaptive immunity (46–48). Antigen presenting cells (APCs) are a type of immune cells that can ingest, process and present antigens to T lymphocytes, and play an important role in immune response. *CD82* and other tetraspanins were previously found in APCs (17, 49). To investigate the effect of *CD82* on pathogen recognition and antigen presentation, we performed bacterial agglutination assays using r*LcCD82a-LEL*. The results showed that in the groups to which r*LcCD82a-LEL* was added, the bacteria all appeared to agglutinate and cluster. *CD82* has been shown to be actively recruited to phagosomes containing fungi (*Cryptococcus neoformans*, *Candida albicans* and *Aspergillus fumigatus*), Gram-positive bacteria (*Staphylococcus aureus*), or Gram-negative bacteria (*Escherichia coli*). And *CD82* overlaps with the endocytic pathway of lipopolysaccharide (LPS), suggesting that *CD82* is related to the presentation of antigen-associated molecules (26). Moreover, previous studies have found that there are antimicrobial proteins and peptides that can agglutinate bacteria. Antimicrobial proteins in saliva cause bacteria in the mouth to agglutinate and act as the first barrier of defense against pathogens (50). Eosinophil cationic protein (ECP) is a secreted protein with strong antimicrobial effects, and it has been reported to have bacterial agglutination activity against Gram-negative bacteria in a concentration range close to the minimal inhibitory concentration, suggesting that agglutination of bacteria plays an essential role in the bactericidal process (51, 52). Based on the above evidence, we reckon that *LcCD82a* is involved in the phagocytosis and antigen presentation of *P. plecoglossicida*, and can agglutinate invading pathogens to inhibit and kill them.

## 5 Conclusion

The spatial and temporal expression patterns of *LcCD82a* in the kidney cell line and individual specimens of the fish were characterized. Two transcript variants, *LcCD82a-L* and *LcCD82a-S*, were identified with differential transcriptional patterns before and after infection with *P. plecoglossicida*, suggesting a potential association with post-infection immune regulation. Further investigation into the molecular functions of the transcript variants *LcCD82a-L/S* and the simulated peptide *LcCD82-LEL* revealed that *LcCD82a* plays a critical role as a membrane protein, involved in extracellular bacterial recognition and aggregation. These findings would be useful for understanding the molecular mechanism of VWND in large yellow croaker.

## Data availability statement

The original contributions presented in the study are included in the article/**Supplementary Material**, further inquiries can be directed to the corresponding author/s.

## Ethics statement

Ethical approval was not required for the studies on humans in accordance with the local legislation and institutional requirements because only commercially available established cell lines were used. The animal study was approved by the Animal Care and Use Committee at the College of Ocean and Earth Sciences, Xiamen University. The study was conducted in accordance with the local legislation and institutional requirements.

## Author contributions

YL: Data curation, Methodology, Validation, Visualization, Writing – original draft, Writing – review & editing. YB: Data curation, Methodology, Writing – review & editing. SC: Data curation, Validation, Writing – review & editing. FP: Supervision, Writing – review & editing. YL: Project administration, Writing – review & editing. HC: Resources, Writing – review & editing. ZZ: Resources, Writing – review & editing. PX: Funding acquisition, Project administration, Resources, Supervision, Writing – review & editing. TZ: Funding acquisition, Project administration, Resources, Supervision, Writing – review & editing.

## Supplementary material

Supplementary Figure 1 The identification of purity of proteins collected from elution samples by SDS-PAGE. Lane 1: protein marker, lane 2: *pET32a-LcCD82a-LEL* bacterial liquid (not induced by IPTG), lane 3: *pET32a-LcCD82a-LEL* bacterial liquid (induced by IPTG), lane 4: Supernatant of *pET32a-LcCD82a-LEL* bacterial solution after sonication, lane 5: The remaining liquid after the supernatant of *pET32a-LcCD82a-LEL* bacterial solution (after sonication) passed through the NTA column, lane 6: Precipitation of *pET32a-LcCD82a-LEL* bacterial solution after sonication, lane 7: The liquid was eluted by 0 mM imidazole, lane 8: the liquid was eluted by 50 mM imidazole, lane 9: the liquid was eluted by 100 mM imidazole, lane 10: the liquid was eluted by 250 mM imidazole, lane 11: the liquid was eluted by 500 mM imidazole.

Supplementary Figure 2 Electron microscopic photographs of *P. plecoglossicida*. Scale bars are 1 µm length in image.
