# Supplementary figures and images for: Molecular characterization, expression pattern and immunologic function of CD82a in large yellow croaker (Larimichthys crocea)
Source: Front Immunol. 2024 Feb 2;15:1301877. doi: 10.3389/fimmu.2024.1301877 (PMC10869527; doi:10.3389/fimmu.2024.1301877)

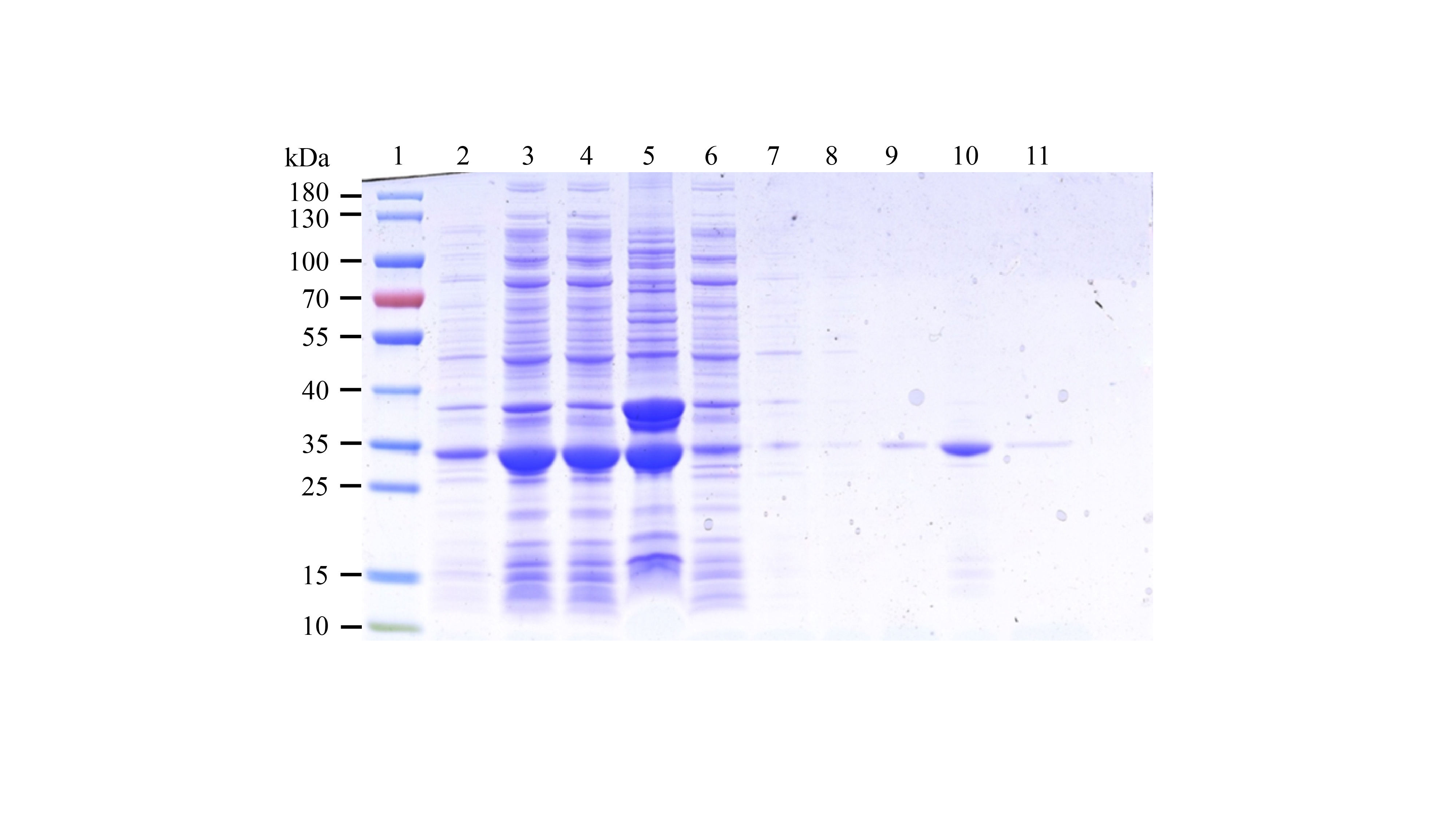

Supplement: Supplementary Figure 1 — The identification of purity of proteins collected from elution samples by SDS-PAGE. Lane 1: protein marker, lane 2: pET32a-LcCD82a-LEL bacterial liquid (not induced by IPTG), lane 3: pET32a-LcCD82a-LEL bacterial liquid (induced by IPTG), lane 4: Supernatant of pET32a-LcCD82a-LEL bacterial solution after sonication, lane 5: The remaining liquid after the supernatant of pET32a-LcCD82a-LEL bacterial solution (after sonication) passed through the NTA column, lane 6: Precipitation of pET32a-LcCD82a-LEL bacterial solution after sonication, lane 7: The liquid was eluted by 0 mM imidazole, lane 8: the liquid was eluted by 50 mM imidazole, lane 9: the liquid was eluted by 100 mM imidazole, lane 10: the liquid was eluted by 250 mM imidazole, lane 11: the liquid was eluted by 500 mM imidazole. [file Image_1.jpeg]

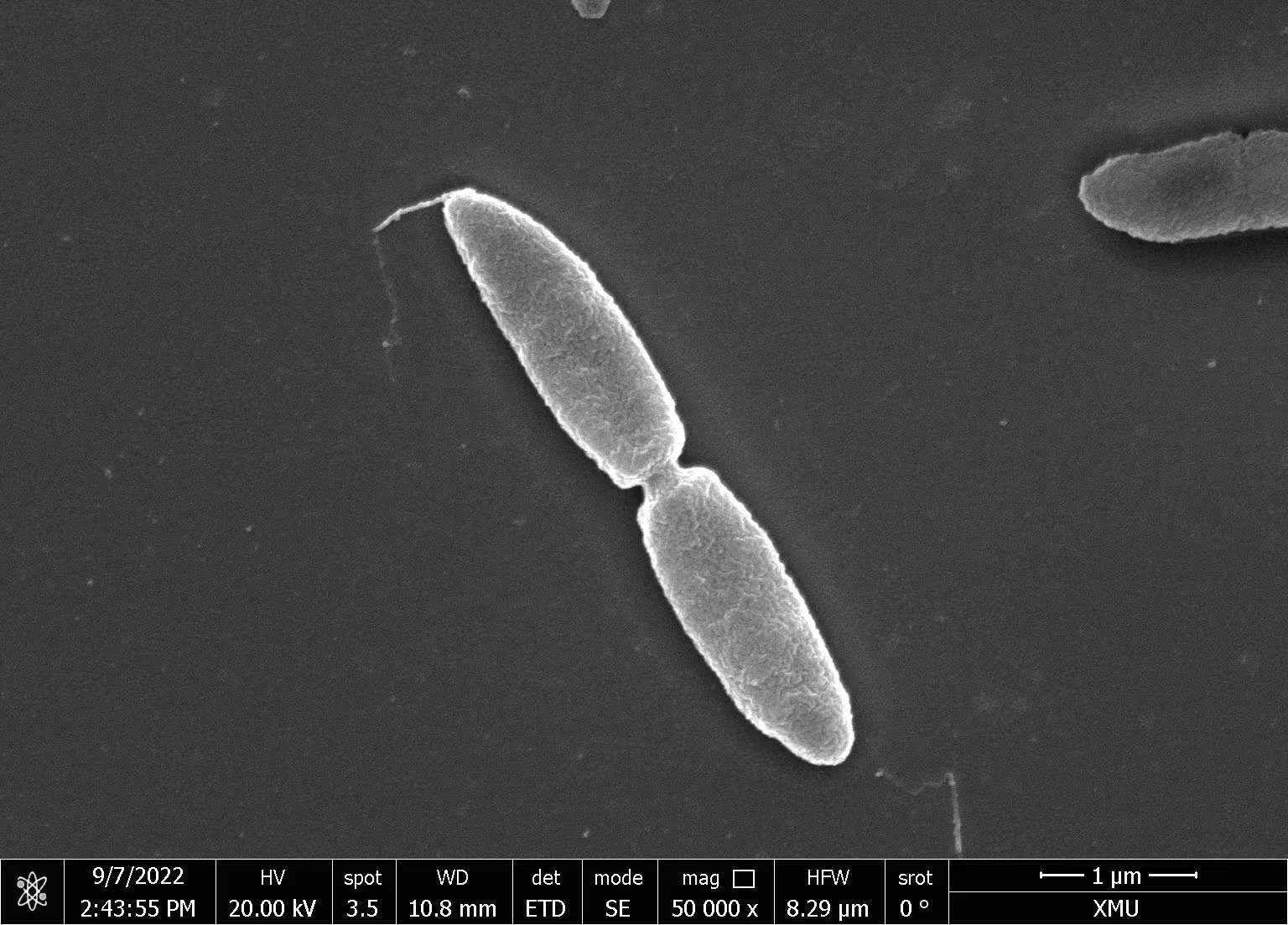

Supplement: Supplementary Figure 2 — Electron microscopic photographs of P. plecoglossicida. Scale bars are 1 µm length in image. [file Image_2.jpeg]
